# Characterization of Key Odorants and Their Content Variation in Sichuan Hotpot Oil With Different Chili Pepper Cultivars by Means of Molecular Sensory Science

**DOI:** 10.1002/fsn3.71098

**Published:** 2025-11-02

**Authors:** Xiaoting Li, Zhengwei Zhang, Bin Li, Qingying Luo, Qianer Chen, Bimal Chitrakar, Gangcheng Wu, Luelue Huang

**Affiliations:** ^1^ School of Food and Drug Shenzhen Polytechnic University Shenzhen Guangdong China; ^2^ College of Food Science and Technology Hebei Agricultural University Baoding China; ^3^ State Key Laboratory of Food Science and Resources, School of Food Science and Technology, National Engineering Research Center for Functional Food, Collaborative Innovation Center of Food Safety and Quality Control in Jiangsu Province Jiangnan University Wuxi Jiangsu China

**Keywords:** aroma activity value, aroma recombination, key odorants, molecular sensory science, Sichuan hotpot oil

## Abstract

In this study, the molecular sensory science method was used to characterize the key odorants in three kinds of Sichuan hotpot oil via HS‐SPME and GC–MS methods. Aroma extract dilution analysis (AEDA), odor activity value (OAV), and aroma recombination were conducted to further analyze its characteristic odor. The AEDA results showed that 26 aroma‐active compounds with higher flavor dilution (FD) values were discovered, while 13 key aroma‐active compounds were identified with OAVs of more than 1. Among them, the main aroma compounds common in the three kinds of Sichuan hotpot oil included nonanal, (*E*)‐2‐octenal, (*E*)‐2‐nonenal, (*E*)‐2‐decenal, (*E*, *E*)‐2,4‐decadienal, and acetic acid. It was worth noting that trans‐β‐inonone, furaneol, and caryophyllene were determined as the special aroma compounds in Xinyidai (XYD) hotpot oil, while 5‐hydroxymethylfurfural was determined in Tianyingjiao (TYJ) hotpot oil. The aroma recombination model resulted in mixed key odorants with OAV ≥ 1, showing excellent sensory acceptability with that of the original sample.

## Graphical abstract

Molecular sensory science method was performed to characterize the key odorants in three kinds of Sichuan hotpot oil. The special aroma compounds were significantly different in three kinds of hotpot oil with different chili pepper cultivars.

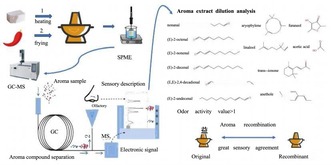

## 1 Introduction

Chili pepper ( *Capsicum annuum* L.) is a diverse genus from Solanaceae, which is globally distributed from Northern to Southwestern regions. It includes around 200 wild and 5 domesticated species (Cirlini et al. 2019). The global production of chili pepper is majorly from China, Vietnam, India, Indonesia, and Brazil, which accounts for more than 80% (with an annual production of over 450,000 tons), reaching the record level of global chili pepper production (de Farias et al. 2020). Chili pepper is an important consumer product, which contains specific components with an attractive pungent and spicy taste while giving many health benefits. It has been a center of interest for food, medicine, and chemical industries (Smith 2015). China boasts a rich diversity of chili varieties, and an extensive range of chili‐based products are available, which is notably exemplified by Sichuan hotpot. Common pepper varieties used for Sichuan hotpot include Shizhuhong, Xinyidai (XYD), Tianyingjiao (TYJ), Denglongjiao, Zidantou, and Yindujiao, among others.

There are various types of chili pepper products in China. Sichuan hotpot is one of such products, which is deeply loved by consumers and is mainly made from edible oils and chili peppers at a certain proportion by stir‐frying at a high temperature to generate a characteristic rich flavor. Therefore, research on the key aroma compounds of hotpot is receiving increasing attention. Sun et al. (2021) analyzed four kinds of commercial Chinese hotpot seasonings by SPME, SAFE, GC‐O‐MS, and PLSR (Sun et al. 2021). Altogether, 53 aroma‐active compounds with high flavor dilution (FD) factors and 17 compounds with the OAV higher than 1 were identified. The recombination and omission experiment showed that the key odorants were identified as 1,8‐cineole, anethole, 2‐acetylthiazole, 2‐furfurylthiol, and (*E*)‐2‐decenal. Yu et al. (2022) employed the comprehensive two‐dimensional GC × GC‐O‐MS to analyze the generation pattern of aroma compounds of hotpot seasoning during boiling and found 23 essential aroma compounds to characterize the major flavor profile of hotpot seasoning during boiling; among them, (*E*)‐2‐heptenal, acetophenone, and phenylacetaldehyde were significantly changed. However, hotpot contains many other substances, such as garlic and bean paste, which in fact comprehensively contribute to the flavor of hotpot. Therefore, it is difficult to clarify the impact of chili pepper on its key odorants. Jiang et al. (2024) showed that processing methods like steaming and stir‐frying significantly influence the volatile compounds in Antarctic krill paste. Similarly, Zhang et al. (2024) emphasized the role of key aroma compounds in star anise essential oil during processing, which included d‐limonene, trans‐α‐citronelene, cineole, trans‐anethole, and wormwood. These studies offer valuable insights into chili peppers' impact on Sichuan hotpot aroma. A study by Yu, Li, et al. (2023) showed that chili pepper varieties significantly affected Sichuan hotpot aroma compounds. However, only limited research on the impact of chili pepper varieties on the hotpot aroma compounds is available. Thus, it is necessary to study the odorant properties of Sichuan hotpot to improve and optimize the flavor.

Molecular sensory science concept was first used by Grosch et al. (1992), which was proven to be a comprehensive approach of combining human sensory perception and instrumental analysis to systematically identify the essential aroma compounds in the bulk of odorless metabolites (Hou et al. 2020). On the basis of identification measurements by GC‐O and GC–MS, together with aroma extract dilution analysis (AEDA), while validating analytical data through OAVs and aroma recombination, the main aroma compounds are characterized. This method has been applied by sensory experts for the characterization of key aroma compounds in various food products. Huang et al. (2021) used molecular sensory science to investigate the key aroma compounds in children's soy sauce. A total of 55 aroma compounds were authenticated by AEDA using GC‐O technology and the OAV results showed 27 key odorants. Yu et al. (2022) found 23 key aroma compounds in hotpot seasoning, and based on the OAV results, acetophenone, (*E*)‐2‐heptenal, and phenylacetaldehyde showed significant changes during boiling. However, to the best of our knowledge, this work constitutes the first research employing concurrent AEDA and OAV approaches to evaluate flavor profiles across these chili cultivars.

Thus, this study used molecular sensory science as an approach to explore the key aroma compounds of Sichuan hotpot. The purpose was to (1) authenticate the aroma compounds of Sichuan hotpot by GC‐O‐MS; (2) accurately quantitate the selected compounds on the basis of AEDA and calculate the OAVs by odor thresholds; (3) construct an aroma recombination model based on the quantitative results and compare them using sensory analysis. The present investigation was believed to provide a theoretical basis for further understanding and improving the aroma compounds of Sichuan hotpot.

## 2 Materials and Methods

### 2.1 Materials

Chili pepper samples with local names Xinyidai (XYD), Tianyingjiao (TYJ), and Moguijiao (MGJ) were obtained from a local market (Shenzhen city, Guangdong, China) in 2023. They were chosen because they were commonly used varieties for hotpot processing. Refined beef tallow and sunflower oil were purchased from the same place.

### 2.2 Chemicals

Standards of n‐alkanes (C_8_–C_40_, ≥ 99%) and benzyl alcohol were obtained from Sigma‐Aldrich (Shanghai, China). Moreover, nonanal (98%), (*E*)‐2‐decenal (95%), (*E*)‐2‐octenal (96%), (*E*)‐2‐nonenal (98%), (*E*, *E*)‐2,4‐decadienal (89%), (*E*)‐2‐undecenal (98%), 5‐hydroxymethylfurfural (99%), furaneol (99%), trans‐β‐ionone (99%), acetic acid (99%), caryophyllene (99%), linalool (98%), 1‐(1H‐pyrrol‐2‐yl)‐ethanone (99%), nonanoic acid (98%), and anethole (98%) were obtained from ANPEL‐TRACE Standard Technical Services Co. Ltd. (Shanghai, China).

### 2.3 Sample Preparation

To investigate the key aroma compounds of Sichuan hotpot oil with three different chili pepper cultivars, only chili peppers were taken to prepare Sichuan hotpot oil. The preparation process was based on previous work (Yu et al. 2022). First, refined beef tallow (50 g) was heated to 150°C and kept it for 5 min. Chili peppers were boiled in water (1:4, w/v) for 10 min and drained thoroughly to obtain boiled chili pepper. Such chili pepper (10 g) was fried in hot oil for 20 min and each oil sample was stored at −18°C for maintaining the original odor characteristics until analysis.

### 2.4 Isolation of the Volatile Compounds

The SPME method was used to collect the volatile components, which were extracted with a 2‐cm long optical fiber (DVB/CAR/PDMS 50/30; Supelco Inc., Bellefonte, PA, USA), following the method described by Xu et al. (2022). Each sample (2 g) was accurately taken in a 20‐mL vial and mixed with 20 μL of standard solution. The sample vial was equilibrated at 80°C for 10 min, and then extracted in an 80°C water bath for 30 min under agitation at 250 rpm. Then, the fiber was injected into the GC–MS port at 250°C for 2 min with a non‐shunt mode.

### 2.5 GC–MS Analysis

GC–MS (Agilent 8890‐5977B, Agilent Technologies, USA) equipment adopts a multi‐function automatic sampling machine (PAL RSI 85, CTC Analytics AG, Switzerland), which works in electronic ionization mode (EI, 70 eV) and is used for separation and analysis of volatile compounds (Xiang et al. 2018). The polar DB‐WAX column (30 m × 0.25 mm × 0.25 μm; Agilent Technologies, USA) was set from 40°C (maintaining for 3 min) to 142°C at 3.5°C/min; ramped at 2°C/min to 150°C; ramped at 3.5°C/min to 177°C; ramped at 6°C/min to 200°C; and finally ramped at 10°C/min to 230°C (maintaining for 3 min). The carrier gas was helium (purity = 99.99%) and the flow rate was 1.0 mL/min. Additional information for mass spectrometry analysis: ion source temperature of 220°C; solvent delay of 5 min; and mass scan range of 35–450 AMU.

### 2.6 GC‐O‐MS Analysis

GC–MS coupled with an Olfactory Detection Port (ODP 2; GERSTEL, Mülheim an der Ruhr, Germany) was employed to analyze the aroma compounds using DB‐WAX column (30 m × 0.25 mm × 0.25 μm). Other conditions were same as that for GC–MS. The olfactory detector port temperature was held at 230°C for all analysis.

For AEDA, the concentrated extract was stepwise split between the olfactory detector port and the MS in the following split ratio: 1:2, 1:4, 1:8, 1:16, 1:32, and so forth. The FD factor was used as the maximum segmentation ratio to clearly detect aroma compounds. The higher the FD factor of aroma compounds, the greater their significance (Song and Liu 2018). According to the same GC conditions mentioned above, this group of extracts were sent to GC‐O‐MS. The diluted aroma compound was analyzed sensorily, which consisted of 20 judges (including 10 females and 10 males; aged between 18 and 40). The panelists participating in the experiment accepted 15 days of training to ensure the identification of all aroma compounds, and each evaluation was repeated in triplicate.

### 2.7 Qualitative and Quantitative Analysis

The obtained mass spectrometry (MS) was compared with MS in the spectral library (Wiley 8 and NIST 14 databases), and the retention time (RT) of the C_8_–C_40_ normal alkane series standard was compared with the retention index of the standard reference compounds for preliminary analysis of the volatile flavor compounds. It was taken as valid when the matching rate between MS and data libraries with an SI (Similar Index) > 85%.

Volatile aroma compounds were quantified using SPME with a PDMS‐coated fiber, followed by GC analysis, while refined sunflower oil was used as the deodorized sample matrix. Benzyl alcohol was used as an internal standard with the recovery yield of 96.86% for the quantification of aroma compounds, while authentic standard calibration curves (external standards) were used to calculate the concentration of each compound. Firstly, the volatile compounds obtained from SPME were quantified (semi‐quantitative analysis) by dividing the peak areas of the compounds of interest by the peak area of the benzyl alcohol as an internal standard (Ozkara et al. 2019). Then, for compounds with available authentic standards, matrix‐matched calibration curves were established using five concentrations of each standard prepared in a deodorized sample matrix. The equilibration time was determined prior to analysis. All standards and samples were extracted and desorbed under strictly identical conditions, facilitated by an SPME auto‐sampler to ensure reproducibility. Peak areas were plotted against concentration to generate linear calibration curves. Concentrations in samples were determined by direct comparison to these external standard curves and were expressed in μg/kg (IOFI 2010, 2011, 2012).

### 2.8 Determination of the Aroma Active Compounds and OAVS

AEDA's results showed that the aroma‐active compounds with FD ≥ 8 were quantitatively analyzed according to the standard curves. Aroma is known to be related to the concentration of aroma compounds, but the odor threshold in the food substrate can also significantly affect the compound's contribution to the overall aroma. Therefore, the odor activity value (OAV) is defined as the odor threshold in water divided by the concentration of the compound, which is used to evaluate the contribution of aroma‐active compounds to the overall aroma of the sample (Yang, Song, et al. 2019). It is generally regarded that compounds with OAVs ≥ 1 have significant contributions to the aroma profiles. All the odor threshold values in water were obtained from previous literature (Ye et al. 2022).

### 2.9 Evaluation of Sensory Properties

The sensory properties of hotpot oil samples were analyzed by quantitative descriptive sensory analysis (QDA). A total of 20 panelists (10 males and 10 females; aged between 18 and 40 years) were employed to assess the sensory properties. All the 20 panelists signed the consent form and received incentives at the end of this study. All panelists were trained using various flavor standards so that they could accurately describe the flavor type and intensity. The intensities of selected odor attributes (chili‐like, cowy, herbaceous, sweet, meaty, fatty) ranged from 0 (imperceptible) to 9 (very strong) on a linear 10‐point dimension. The methods of selection and training panelists were based on Thurer and Granvogl (2016) with some modifications. Each sample (10 g) was heated to meat (60°C–70°C) and homogeneously presented in a coded cup for evaluation. The reference solution in refined sunflower oil was provided for each aroma descriptor. Various compounds with their corresponding odor descriptions were as follows: β‐caryophyllene for chili‐like; (*E*)‐2‐decenal for cowy; linalool for herbaceous; furaneol for sweet; 1‐(1H‐pyrrol‐2‐yl)‐ethanone for meaty; and nonanal for fatty. Each sample was evaluated at least three times independently by each panelist, and average values were recorded.

### 2.10 Aroma Recombination

On the basis of GC‐O‐MS analysis and OAV calculations, the aroma active compounds with OAV > 1 were ultimately identified as the key aroma compounds in this study. The recombination experiment was carried out with all the flavor indexes of OAVs ≥ 1 as the main flavor components at their respective occurring concentrations into the odorless matrix. The recombinant was evaluated with the original one in parallel by panelists according to whether it was consistent with the original sample, while the aroma evaluation method was the same as outlined above.

### 2.11 Statistical Analysis

The experimental results were expressed as the mean ± standard deviation (SD) using Microsoft Office Excel 2019. QDA sensory evaluation and GC–MS data were analyzed by one‐way analysis of variance using SPSS 23.0 software (SPSS Inc., Chicago, IL, USA) at a 95% confidence level (*p* < 0.05). The heatmap of volatile compounds and the spider diagram of sensory evaluation were plotted by Origin 2021 (Origin Lab Corporation, Northampton, MA).

## 3 Results and Discussion

### 3.1 Volatile Aroma Compounds in Sichuan Hotpot Oil

**FIGURE 1:**
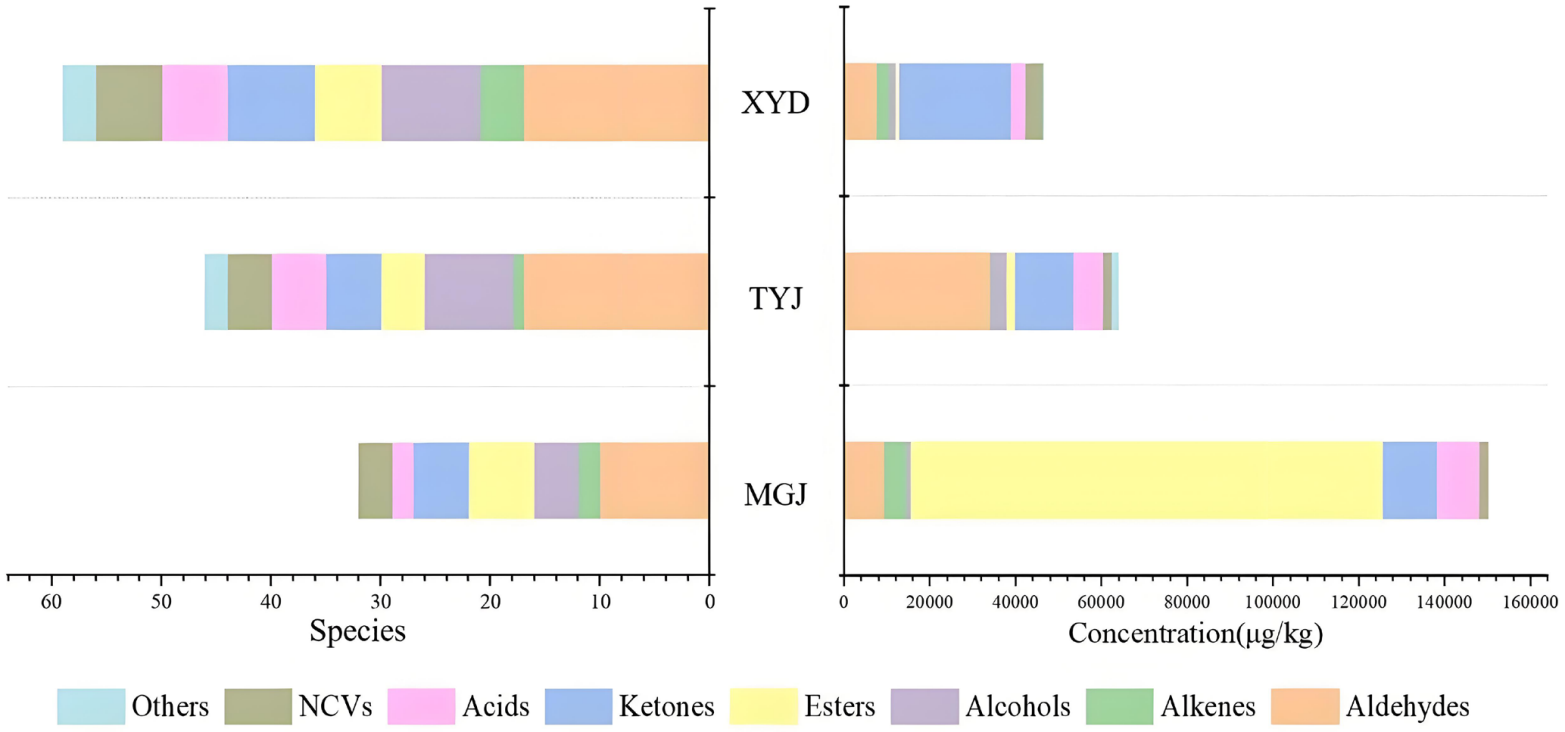
The stack diagram of volatile compounds of Sichuan hotpot oil with different chili peppers.

GC–MS was used to identify the volatile aroma compounds using an external standard (n‐alkanes, namely C_8_–C_40_) and semi‐quantitatively calculated by an internal standard. As shown in Figure 1, 54 volatile compounds were confirmed in Sichuan hotpot oil, where 7 chemical categories were set to classify them, including 16 aldehydes, 4 alkenes, 7 alcohols, 8 esters, 7 ketones, 5 acids, 4 nitrogen‐contained volatiles (NCVs), and 3 others. This indicated that hotpot oil samples consisted of abundant flavor compounds. There were 49, 42, and 30 volatile aroma compound varieties present in XYD, TYJ, and MGJ samples, respectively. The XYD hotpot oil detected the highest ketone content; the TYJ hotpot oil detected the highest aldehyde content; and the MGJ hotpot oil detected the highest ester content, indicating significant differences (*p* < 0.05) in volatile compound content among the three types of hot pot oils. Apart from ketones, aldehydes, and esters, alcohols, acids, and alkenes were considered the key volatile components, contributing to the potent aroma in hot pot oil, which played an important role in the aroma characteristics of commercial hotpot (Sun et al. 2021).

**FIGURE 2:**
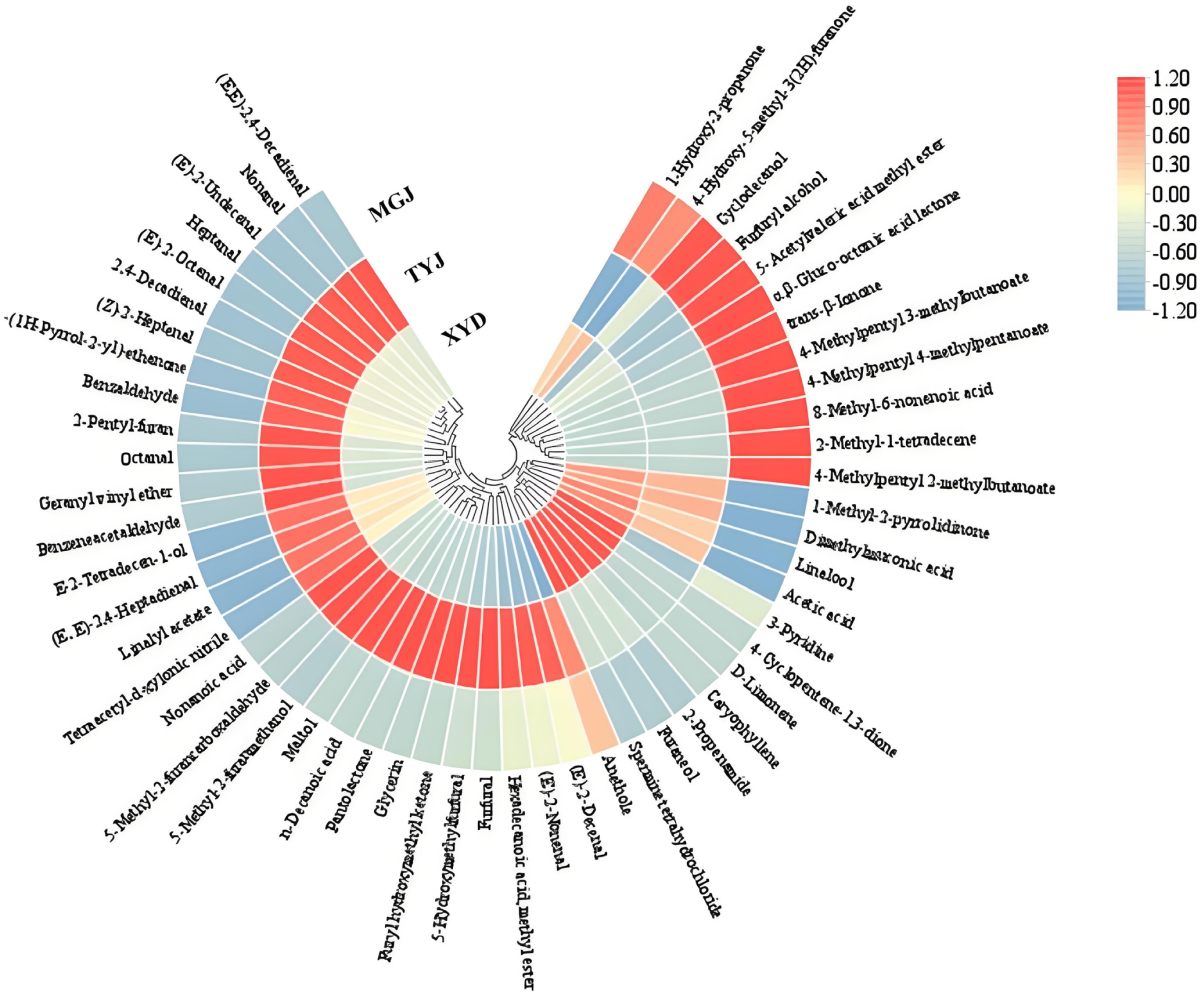
Heat map analysis of volatile compounds in Sichuan hotpot oil with different chili peppers.

Figure 2 shows the overall profile of volatile compounds in hotpot oil with different chili peppers, with colored boxes showing the hierarchical clustering analysis (HCA). For inhibiting the bulk of volatile compounds from more to less, the intensity of color (on the basis of normalizing concentrations) ranged from maximum value (red) to minimum value (blue) (Florentino‐Ramos et al. 2019). The heatmap analysis showed that there were differences in the content of volatile compounds in the hotpot oil samples. According to the dendrogram, the volatile compounds of three hotpot oil samples could be classified into four categories. Among them, the compound occurring at the highest concentration was determined as 4‐methylpentyl 3‐methylbutanoate (MGJ, 92,892 μg/kg).

Aldehydes are important volatile compounds for hotpot oils. A significant difference (*p* < 0.05) in aldehyde content was observed among three types of hot pot oil samples. Among them, the TYJ sample showed the highest content of aldehydes (33,088.51 μg/kg), which accounted for 48.67% of the total aroma compounds, while XYD and MGJ showed 15,828.28 μg/kg (29.30%) and 9109.31 μg/kg (5.94%), respectively. The types of aldehydes detected in the three hotpot oil samples were similar, while the relative contents of them showed evidently different results. For example, benzaldehyde was detected as the main volatile compound in the XYD sample (8626.21 μg/kg), while 5‐hydroxymethylfurfural was the highest volatile compound in TYJ (12,257.92 μg/kg) and MGJ (2719.11 μg/kg). This was consistent with previous research that the differences in aldehyde content were mainly due to the divergence of antioxidant activity caused by differences in capsaicinoids content among various chili pepper cultivars (Yu, Li, et al. 2023). Three hotpot oil samples were made from different cultivars of chili pepper, which might result in differences in capsaicinoids content, further leading to significant differences in the types and contents of aldehydes. The dominant aldehydes in the three hotpot oil samples were furfural, benzene acetaldehyde, (*E*)‐2‐decenal, and 5‐hydroxymethylfurfural, which were associated with a green and fatty odor. They were also the largest volatile compounds determined in fried pepper oil (Sun et al. 2020). Aldehydes showed a low odor threshold, contributing well to the overall flavor. The aldehydes in hotpot oil mainly came from refined tallow. On the one hand, the tallow was heated and deodorized during the refining process, which caused the cells to be heated and broken. The internal fatty acids, when heated and came into contact with oxygen in the presence of lipoxygenase, were oxidized to form hydroperoxides, which further decomposed to form aldehydes and other volatile compounds. On the other hand, refined tallow reacted with chili peppers during high‐temperature frying, producing aldehydes via Strecker degradation (Ivanova‐Petropulos et al. 2015; Liu et al. 2019).

Ketones were the second main volatile compounds in these three kinds of hot pot oil samples, and a significant difference (*p* < 0.05) in ketone contents was observed among them. The ketone content in the XYD sample was the highest (26,039.18 μg/kg), while such content in MGJ was the lowest (12,611.76 μg/kg). The types of ketones detected in the three hotpot oil samples were also different; the XYD sample detected 8 types of ketones, while TYJ and MGJ detected only 4 types of ketones. Among them, furyl hydroxymethyl ketone, furaneol, and 4‐hydroxy‐5‐methyl‐3 (2H)‐furanone were common ketones. A special volatile compound, called 2,3‐dihydro‐3,5‐dihydroxy‐6‐methyl‐4H‐pyran‐4‐one, was detected in the XYD sample (24,182.30 μg/kg), which was the main reason for the high ketone content. It was determined to be the main volatile component of aromatic rapeseed oil (Zhang et al. 2023). The main reason for the formation of ketones was probably the oxidative decomposition of unsaturated fatty acids and the decomposition of amino acids (Zhang et al. 2018). Most of the ketones had relatively high thresholds and were the main substances that caused the formation of meaty flavor. Thus, the differences in the content and variety of ketones in various hotpot oil samples might lead to differences in their flavor.

Significant differences in esters content were observed among three types of hot pot oil samples (*p* < 0.05). The esters content in the MGJ sample was the highest (109,904.21 μg/kg), while such content in the TYJ sample was the lowest (9109.31 μg/kg). The types of esters detected in the three hotpot oil samples also showed differences. Among the 10 types of esters, only 4‐methylpentyl 2‐methylbutanoate and hexadecanoic acid methyl ester were detected as common volatile compounds in the three hot pot oil samples. Esters were the volatile compounds dominating in the MGJ sample, accounting for 67.47% of the total aroma compounds content, mainly due to the presence of 4‐methylpentyl 3‐methylbutanoate and 4‐methylpentyl 2‐methylbutanoate. Previous studies have shown that the above‐mentioned esters gave fruit flavor to chili pepper. Esters are important aroma compounds produced by the esterification of fatty alcohol and short‐chain acid in hot pot oil, contributing the main volatile compounds in chili pepper (Li et al. 2022). However, the threshold value of esters is high; thus, their contribution to the overall flavor of food is generally small.

Acids, alcohols, alkenes, NCVs, and other volatile compounds accounted for a small part of the hotpot oil; however, they might still affect the overall flavor. Their contents in three hotpot samples also showed significant differences (*p* < 0.05). Among them, acids had a greater contribution to the overall flavor due to their lower threshold. Previous studies showed that the main volatile substance in red and bell pepper‐flavored olive oil was acetic acid, which was probably produced by fermentation during the dehydration of peppers (Gogus et al. 2015).

### 3.2 Aroma‐Active Compounds in Sichuan Hotpot

Ample information indicated that only a small fraction of abundant volatile compounds presented in the food matrix actually has contributions to its overall aroma characteristics and aroma perception (Vidal et al. 2019). Such a small fraction detected by olfaction is often referred to as aroma‐active compounds, which makes an important contribution to the choice, preference, and acceptance of consumers. A useful tool to screen the aroma‐active compounds, known as AEDA, was performed to gain deeper insights into the aroma characteristics and the intensities of key aroma compounds.

The AEDA method through GC‐O‐MS analysis showed 26 aroma‐active compounds with FD values ranging from 2 to 32 with an appreciable odor intensity. As shown in Table 1, six kinds of aroma‐active compounds in three hotpot oil samples were detected, including 12 aldehydes, 5 ketones, 2 alkenes, 2 acids, 2 alcohols, 2 esters, and 1 NCVs. The number of aroma‐active compounds found in XYD, TYJ, and MGJ was 23, 23, and 12, respectively, indicating more such compounds in the first two samples (XYD and TYJ) with higher FD values, while a slightly different compound variation occurred. However, the MGJ sample showed fewer aroma‐active compounds with lower FD values, while the key aroma‐active compounds with higher FD values were mainly concentrated in aldehydes. The possible reasons might be that MGJ chili pepper had a higher capsaicinoids content than XYD and TYJ; thus, the former had a stronger antioxidant activity, slowing down the interaction process of lipid oxidation and the Maillard reaction, which might result in fewer flavor‐active compounds except aldehydes. Taking into consideration the FD values, the following 15 volatile compounds had higher FD values (≥ 8), which might play an essential role in the overall aroma characteristics of Sichuan hotpot oil samples (Zhang et al. 2017): nonanal, (*E*)‐2‐octenal, (*E*)‐2‐nonenal, (*E*)‐2‐decenal, (*E*,*E*)‐2,4‐decadienal, 5‐hydroxymethylfurfural, (*E*)‐2‐undecenal, caryophyllene, anethole, linalool, trans‐β‐ionone, furaneol, acetic acid, nonanoic acid, and 1‐(1H‐pyrrol‐2‐yl)‐ethanone.

**TABLE 1:** Aroma‐active compounds determined by AEDA in Sichuan hotpot oil.

Compounds	RI^a^	Odor description^b^	Identification^c^	FD factor^d^		
				XYD	TYJ	MGJ
Aldehydes						
Octanal	1262	Fat, soap, lemon	MS, RI, O	4	ND	ND
Nonanal	1390	Fat, green	MS, RI, O	16	16	ND
(E)‐2‐Octenal	1437	Green, nut, fat	MS, RI, O	8	16	32
Furfural	1472	Almond, sweet	MS, RI, O	4	4	4
Benzaldehyde	1492	Caramel, fruit	MS, RI, O	4	4	ND
(E)‐2‐Nonenal	1507	Cucumber, fat	MS, RI, O	32	32	16
5‐Methyl‐2‐furancarboxaldehyde	1575	Sweet	MS, RI, O	ND	8	ND
Benzene acetaldehyde	1648	Sweet, floral	MS, RI, O	2	4	4
(E)‐2‐Decenal	1640	Tallow	MS, RI, O	16	32	16
(E)‐2‐Undecenal	1747	Fat	MS, RI, O	16	16	ND
(E, E)‐2,4‐Decadienal	1820	Fat	MS, RI, O	16	32	32
5‐Hydroxymethylfurfural	2526	Woody	MS, RI, O	4	2	4
Alkenes						
Caryophyllene	1580	Woody, spicy	MS, RI, O	32	ND	ND
Alcohols						
Linalool	1516	Floral, lavender	MS, RI, O	32	8	ND
5‐Methyl‐2‐furanmethanol	1729	Mint, herbaceous	MS, RI, O	ND	4	ND
Esters						
Anethole	1820	Anise	MS, RI, O	32	16	4
Linalyl acetate	2176	Floral, sweet	MS, RI, O	2	4	ND
Hexadecanoic acid, methyl ester	2202	Fat, orris	MS, RI, O	4	2	2
Ketones						
1‐Methyl‐2‐pyrrolidinone	1670	Green	MS, RI, O	4	4	ND
Trans‐β‐Ionone	1923	Floral	MS, RI, O	32	4	ND
Furaneol	2038	Caramel	MS, RI, O	32	4	2
4‐Hydroxy‐5‐methyl‐3 (2H)‐furanone	1087	Caramel, roast	MS, RI, O	4	ND	4
2,3‐Dihydro‐3,5‐dihydroxy‐6‐methyl‐4H‐pyran‐4‐one	2240	Roast, green	MS, RI, O	4	2	ND
Acids						
Acetic acid	1475	Sour	MS, RI, O	16	16	32
Nonanoic acid	2118	Green, fat	MS, RI, O	ND	8	ND
NCVs						
1‐(1H‐Pyrrol‐2‐yl)‐ethanone	1968	Roast	MS, RI, O	8	8	ND

Abbreviation: ND, not detected.

^a^ Retention index: compounds were identified on a TB‐WAX column by comparison to library standard value.

^b^ Odor description: odors were perceived at the sniffing port.

^c^ Identification based on NIST 14 mass spectral database (MS); published RIs; published odor descriptions.

^d^ Flavor dilution factor.

From the comparison of three samples, the aroma‐active compounds with the highest FD (32) factors were identified as (*E*)‐2‐octenal in MGJ sample (green), (*E*)‐2‐nonenal in XYD and TYJ (fat), (*E*)‐2‐decenal in TYJ (tallow), (*E*,*E*)‐2,4‐decadienal in TYJ and MGJ (fat), caryophyllene in XYD (spicy), anethole in XYD (anise), linalool in XYD (floral), trans‐β‐ionone in XYD (floral), furaneol in XYD (caramel), and acetic acid in MGJ (sour). Aldehydes are significant aroma‐active compounds with the characteristics of fatty, green, and tallow and are important contributors to beef flavor. On the basis of AEDA results, most of the aroma‐active compounds determined in the TYJ sample were the same as those found in the XYD sample with lower FD values, which was consistent with the quantitative analysis results described above. These 15 aroma‐active compounds with higher FD values were generally the main compounds responsible for the principal aroma of hotpot oil.

Unsaturated fatty acids are susceptible to oxidation via autoxidation, enzyme‐mediated catalysis, or photooxidation. For flavor generation in Sichuan hotpot oil, autoxidation represents the most critical pathway. This process follows a classic free radical mechanism comprising three stages: initiation (radical formation), propagation (radical chain reactions), and termination (production of inactive species via radical combination or disproportionation) (Musakhanian et al. 2022). For example, the characteristic flavor profile of Sichuan hotpot oil develops through thermally driven reactions. 2,4‐Decadienal primarily originated from autoxidation of ω‐6 fatty acids (e.g., linoleic acid), undergoing initiation, propagation, and β‐scission of hydroperoxides. Moreover, trans‐β‐ionone formed mainly via acid‐catalyzed degradation of chili carotenoids, with potential Maillard‐assisted routes through nor isoprenoid retro‐aldol condensation. In addition, furaneol was synthesized via Maillard reactions, where pentose sugars underwent Strecker degradation to 2‐deoxyribosone, followed by enolization/cyclization to furaneol. For capsaicinoids, they degraded at a temperature higher than 160°C to yield vanillin and C₆–C₁₀ aldehydes (e.g., 2,4‐decadienal) through alkyl chain scission (Wang et al. 2023). These compounds constituted key contributors to the characteristic flavor profile of Sichuan hotpot oil.

### 3.3 OAVs of Aroma‐Active Compounds

The limitation of the AEDA method is that the molecular interaction between odorants and the food matrix is not considered, which has a considerable effect on aroma release, leading to a different response of the human olfactory receptors (Matheis and Granvogl 2016). Moreover, it is not true that the higher the content of volatile compounds, the stronger the odor perception by human sensory organs; yet it is related to the odor threshold (Greger and Schieberle 2007). Therefore, it is necessary to conduct quantitative analysis through aroma dilution analysis and determination of the corresponding odor threshold. OAV can reasonably estimate a single odorant on the overall aroma; thus, it has been widely applied in key odorant research. Generally, compounds with OAV ≥ 1 play a vital role in the overall aroma of foods (Greger and Schieberle 2007).

Quantitative analysis of aroma‐active compounds with FD value ≥ 8 was conducted along with GC–MS analysis by constructing standard curves, which were also used to establish aroma recombination models. To gain knowledge on the potential odor of the aroma‐active compounds to the characteristic aroma of Sichuan hotpot oil, their odor thresholds in water (OAVs) as well as concentrations were listed in Table 2. A total of 13 aroma‐active compounds yielded OAVs greater than 1, stating that these compounds had major contributions to the overall aroma profile of Sichuan hotpot oil. Among them, the types and contents of flavor compounds in three kinds of hot pot oil samples were different. 12, 10, and 6 flavor compounds were detected in XYD, TYJ, and MGJ hotpot oil samples, respectively. These compounds were present at concentrations ranging from 49.70 μg/kg in TYJ (linalool) to 4560.86 μg/kg in TYJ (nonanoic acid). There were 4 compounds, including nonanal (TYJ, OAV = 1509.56), (*E*)‐2‐octenal (MGJ, OAV = 1132.57), (*E*)‐2‐nonenal (XYD, OAV = 6580.96; TYJ, OAV = 5375.75; MGJ, OAV = 2541.31), and (*E*, *E*)‐2,4‐decadienal (XYD, OAV = 7836.65; TYJ, OAV = 19,683.51; and MGJ, OAV = 49739.33), deserving high attention due to high OAVs (OAVs > 1000). Most of these compounds are also key aroma compounds in spicy products (Yu, Zhang, et al. 2023). Twenty aroma‐active compounds critical to the seasoning's profile, including linalool and (*E*)‐2‐decenal, were confirmed via recombination‐omission tests. Sensory analysis revealed the aldehydes imparting fatty/meaty notes, sulfurous characteristics, and terpenes enhancing spice‐like aromas. In addition, compared to Thai fried chili pastes heated for 50 min at 100°C, the 25‐min sample exhibited distinct predominant odorants, namely 3‐vinyl‐4H‐1,2‐dithiin, allyl methyl disulfide, and allyl methyl trisulfide. Notably, dimethyl trisulfide and diallyl disulfide demonstrated the highest OAVs at 25 min. This indicated that thermally derived volatiles were generated via Maillard/Strecker reactions or sulfur‐compound degradation, which dominated the aroma profile of briefly heated pastes (Rotsatchakul et al. 2008). It was worth noting that trans‐β‐inonone, furaneol, and caryophyllene were detected as key aromas only in XYD hotpot oil, and 5‐hydroxymethylfurfural was detected only in TYJ hotpot oil. However, the key aroma‐active compounds of MGJ hotpot oil showed less quantity and fewer differences in type, which was consistent with the AEDA results. This might be the main reason for the significant flavor differences among the three hotpot oil samples. In addition, (*E*)‐2‐decenal, (*E*)‐2‐undecenal, trans‐β‐ionone, caryophyllene, anethole, linalool, and acetic acid showed higher OAVs (> 20), which were considered to be the main sources of meaty, herbaceous, sweet, and spicy aromas, as explained in a previous study (Lu et al. 2019). Thus, the cultivar of chili pepper certainly affected the types and contents of key odorants in Sichuan hotpot.

**TABLE 2:** Concentrations, odor thresholds, and OAVs of key aroma‐active compounds in Sichuan hotpot oil.

No.	Compounds	Standard curves	*R* ^2^	Odor threshold (μg/kg)	Concentrations (μg/kg)			OAV		
					XYD	TYJ	MGJ	XYD	TYJ	MGJ
1	Nonanal	*Y* = 12,329× + 490,100	0.9982	1.1	967.89 ± 58.42^b^	1660.52 ± 69.11^a^	470.64 ± 30.52^c^	879.90	1509.56	427.86
2	(E)‐2‐Octenal	*Y* = 10,389× + 1,840,000	0.9982	3	145.61 ± 48.40^d^	722.24 ± 29.65^b^	3397.70 ± 73.95^a^	48.54	240.75	1132.57
3	(E)‐2‐Nonenal	*Y* = 15,321×−627,990	0.9974	0.19	1250.38 ± 74.77^a^	1021.39 ± 4.36^b^	482.85 ± 0.65^d^	6580.96	5375.73	2541.31
4	5‐Hydroxymethylfurfural	*Y* = 20,063×−4,838,600	0.9942	1110	ND	1521.10 ± 163.05^a^	ND	ND	1.37	ND
5	(E)‐2‐Decenal	*Y* = 16,309×−906,090	0.9990	17	952.36 ± 54.94^d^	3394.55 ± 99.63^a^	1381.71 ± 53.19^c^	56.02	199.68	81.28
6	(E)‐2‐Undecenal	*Y* = 15,137×−126,570	0.9983	1.4	514.46 ± 66.49^c^	708.59 ± 38.59 ^b^	ND	367.47	506.13	ND
7	(E,E)‐2,4‐Decadienal	*Y* = 79,046× + 2,802,900	0.9902	0.027	656.47 ± 43.88^c^	531.45 ± 93.74^d^	1342.96 ± 3.05^a^	7836.65	19683.51	49739.33
8	Trans‐β‐Ionone	*Y* = 12,129×−105,580	0.9953	3.5	150.54 ± 39.46	ND	ND	43.01	ND	ND
9	Furaneol	*Y* = 15,169× + 65,883	0.9932	22.3	181.77 ± 49.05	ND	ND	8.15	ND	ND
10	Caryophyllene	*Y* = 13,242× + 3,361,500	0.9953	64	2903.37 ± 44.25^b^	ND	ND	45.37	ND	ND
11	Anethole	*Y* = 14,037× + 398,640	0.9905	15	919.06 ± 6.32^b^	575.38 ± 19.62^c^	ND	61.27	38.36	ND
12	Linalool	*Y* = 16,193× + 64,160	0.9985	2.2	136.35 ± 37.93^a^	49.70 ± 12.64^c^	ND	61.98	22.59	ND
13	Acetic acid	*Y* = 10,124× + 2,964,700	0.9914	50	2272.37 ± 165.68^d^	2445.64 ± 147.59^b^	14191.51 ± 35.97^a^	45.45	48.91	283.83
14	Nonanoic acid	*Y* = 16,460×−3,040,100	0.9977	4600	ND	4560.86 ± 13.30^a^	ND	ND	0.99	ND
15	1‐(1H‐pyrrol‐2‐yl)‐Ethanone	*Y* = 13,524 + 496,988	0.9932	58595.25	1593.29 ± 46.43^a^	1537.12 ± 104.42^b^	ND	0.03	0.03	ND

*Note:* Data are expressed as mean ± SD (*n* = 3). The values followed by different letters in a line are significantly different. Data marked with different letters show significant differences at *p* < 0.05 using SPSS 23.0 software. Different lowercase letters represent a statistically significant difference in each line. Odor thresholds in water (OAVs) are referenced from the literature. OAVs are calculated by dividing the concentrations by the odor threshold.

It was shown that most FD values were basically consistent with OAV, whereas some exceptions existed as well. For instance, nonanoic acid showed a higher FD value in the XYD sample (FD = 8), while the OAV was less than 1 (OAV = 0.03). The unexpected result was most likely explained when performing the OAV approach. It is on the basis of the assumption that the overall aroma characteristics of foods are only a simple superposition of the aroma attributes of each aroma substance, while the actual aroma compounds have synergistic or antagonistic effects in the overall aroma contribution. Moreover, the OAV method suggests that the aroma intensity of each compound increased linearly with increasing concentration (Zhang et al. 2017). In fact, the response relationship between the concentration logarithm of each aroma compound and the aroma intensity generally follows an S‐shaped non‐linear curve. Besides, the psychological measurement of each aroma compound should also be noteworthy (Yang, Zheng, et al. 2019).

### 3.4 Aroma Recombination

On most occasions, any aroma‐active compounds in food endure the typical odorants of the food itself. Moreover, it is considered that the prediction of the overall aroma of complicated mixtures is difficult (Greger and Schieberle 2007). Thus, performing an aroma recombination was an essential step to further validate the contribution of key aroma compounds to the overall aroma of Sichuan hotpot oil. A total of 12 standards of aroma compounds with OAVs ≥ 1 (same concentrations achieved by quantitative analysis, Table 2) were admixed in odorless matrices, constituting the recombination models. Compared to XYD and MGJ samples, TYJ contained the highest levels of nonanal, (*E*)‐2‐decenal, and nonanoic acid, whereas trans‐β‐ionone, furaneol, and caryophyllene were undetected. Conversely, XYD exhibited the highest concentrations of (*E*)‐2‐nonenal, linalool, and 1‐(1H‐pyrrol‐2‐yl)‐ethanone, though nonanoic acid and 5‐hydroxymethylfurfural were absent. Notably, only six key aroma‐active compounds were detected in MGJ, with (*E*)‐2‐octenal, acetic acid, and (*E*,*E*)‐2,4‐decadienal being predominant.

**FIGURE 3:**
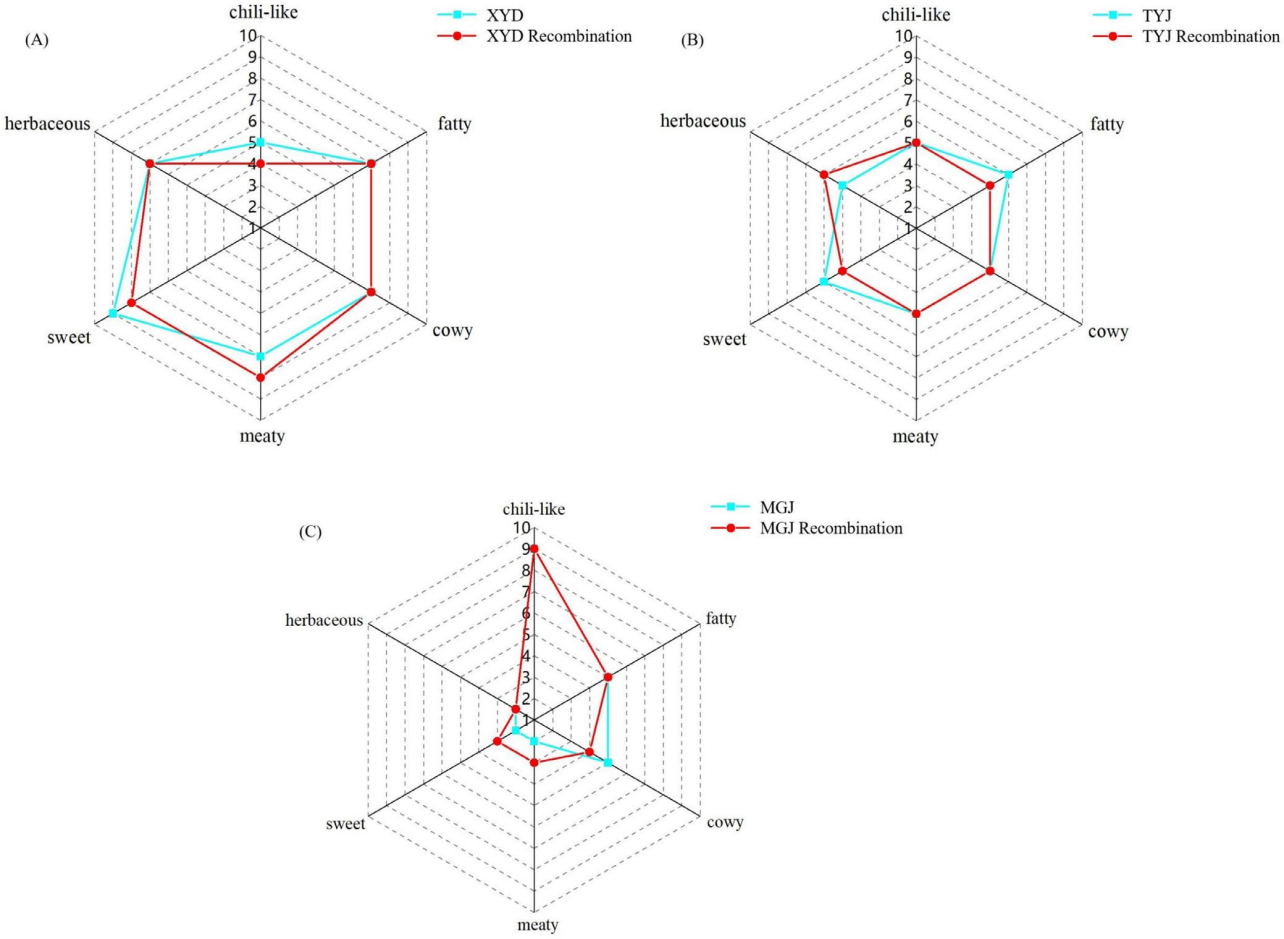
Aroma profile of Sichuan hotpot oil compared with aroma recombination. (A) XYD and XYD recombination. (B) TYJ and TYJ recombination. (C) MGJ and MGJ recombination. The mean rating of each note was compared using a *T*‐test and the result showed no significant differences (*p* > 0.05).

The recombinants were then compared with the original samples to determine the differences or similarities. As illustrated in Figure 3, the aroma profiles of recombinants showed eminent agreement apart from some minor differences in chili‐like, sweet, meaty, and herbaceous flavor, which were characteristics of the original one and were most likely due to masking, inhibition, or a synergistic effect between the compounds with OAV greater or less than 1. The intensities of the fatty and cowy sensory attributes did not differ significantly between the samples. The flavor composition of the XYD sample, however, was mainly defined by sweet, meaty, and herbaceous notes. Conversely, the MGJ sample featured a significantly more intense chili‐like flavor. A comprehensive molecular understanding of the interactions among pungency, chili‐like, sweet, meaty, and herbaceous flavors, while volatile constituents within Sichuan hotpot oil, was currently limited. In contrast, several recent investigations explored the synergistic potential of pungent and sweet sensations in augmenting savory taste perception. It was suggested that the taste thresholds of sweet could be reduced by low concentrations of capsaicin (Wang et al. 2023). Contrary to some expectations, our findings revealed an inverse relationship between pungency intensity and perceived sweetness, wherein an increased pungent compound concentration corresponded to diminished sweetness perception (Figure 3). The precise causes of this phenomenon remain to be elucidated.

There was no significant difference (*p* > 0.05) observed among the aroma recombinants, the original samples, and the overall aroma similarity reached 9.5 of 10 points, suggesting that this study successfully identified the key aroma compounds of Sichuan hotpot oil. Therefore, the key aroma compounds in XYD hotpot oil were determined as nonanal, (*E*)‐2‐octenal, (*E*)‐2‐nonenal, (*E*)‐2‐decenal, (*E*)‐2‐undecenal, (*E*, *E*)‐2,4‐decadienal, caryophyllene, anethole, trans‐β‐ionone, furaneol, linalool, and acetic acid. The key odorants in TYJ hotpot oil were determined as nonanal, (*E*)‐2‐octenal, (*E*)‐2‐nonenal, (*E*)‐2‐decenal, (*E*)‐2‐undecenal, (*E*, *E*)‐2,4‐decadienal, 5‐hydroxymethylfurfural, anethole, linalool, and acetic acid. The key odorants in MGJ hotpot oil were determined as nonanal, (*E*)‐2‐octenal, (*E*)‐2‐nonenal, (*E*)‐2‐decenal, (*E*, *E*)‐2,4‐decadienal, and acetic acid.

## 4 Conclusion

In conclusion, this study used molecular sensory science to characterize the key odors of Sichuan hotpot oil. SPME‐GC–MS was used to identify and quantify 54 volatile compounds in Sichuan hotpot oil. Twenty‐six compounds with higher FD values were identified as aroma‐active compounds based on GC‐O‐MS and AEDA methods. Further, through the application of OAV calculations, 13 compounds with OAV greater than 1 were considered to be the most significant odorants of Sichuan hotpot oil. They included nonanal, (*E*)‐2‐octenal, (*E*)‐2‐nonenal, (*E*)‐2‐decenal, (*E*, *E*)‐2,4‐decadienal, (*E*)‐2‐undecenal, caryophyllene, linalool, 5‐hydroxymethylfurfural, anethole, trans‐β‐ionone, furaneol, and acetic acid. Among them, trans‐β‐ionone, furaneol, and caryophyllene were determined to be the special aroma compounds in XYD hotpot oil, while 5‐hydroxymethylfurfural was determined to be in TYJ hotpot oil. Aroma recombination studies showed great sensory agreement between the recombinant and original hotpot. This study enriched the knowledge of the key odorants of Sichuan hotpot oil. Follow‐up research may investigate the formation mechanism of key odorants in the process of hotpot, which makes further efforts to comprehend the flavor control and quality improvement of hotpot.

## Author Contributions

**Xiaoting Li:** conceptualization, data curation, formal analysis, methodology, and writing original draft; **Zhengwei Zhang:** data curation, formal analysis, investigation, writing – review and editing; **Bin Li:** data curation, reviewing, and editing of the final manuscript draft; **Qingying Luo:** data curation, validation, as well as reviewing and editing of the final manuscript draft; **Qianer Chen:** data curation and investigation; **Bimal Chitrakar:** data curation and validation; **Gangcheng Wu:** formal analysis and editing of the final manuscript draft; **Luelue Huang:** resources, data curation, funding acquisition, and writing.

## Conflicts of Interest

The authors declare no conflicts of interest.

## Data Availability

The data that support the findings of this study are available from the corresponding author upon reasonable request.

## References

[fsn371098-bib-0001] Cirlini, M. , G. Luzzini , E. Morini , et al. 2019. “Evaluation of the Volatile Fraction, Pungency and Extractable Color of Different Italian *Capsicum annuum* Cultivars Designed for Food Industry.” European Food Research and Technology 245, no. 12: 2669–2678. 10.1007/s00217-019-03378-x.

[fsn371098-bib-0002] de Farias, V. L. , I. M. D. Araujo , R. F. J. da Rocha , D. D. Garruti , and G. A. S. Pinto . 2020. “Enzymatic Maceration of Tabasco Pepper: Effect on the Yield, Chemical and Sensory Aspects of the Sauce.” LWT ‐ Food Science and Technology 127: 109311. 10.1016/j.lwt.2020.109311.

[fsn371098-bib-0003] Florentino‐Ramos, E. , N. Villa‐Ruano , D. Hidalgo‐Martínez , et al. 2019. “1H NMR‐Based Fingerprinting of Eleven Mexican *Capsicum annuum* Cultivars.” Food Research International 121: 12–19. 10.1016/j.foodres.2019.03.025. 31108732

[fsn371098-bib-0004] Gogus, F. , M. Z. Ozel , H. Keskin , D. K. Yanik , and A. C. Lewis . 2015. “Volatiles of Fresh and Commercial Sweet Red Pepper Pastes: Processing Methods and Microwave Assisted Extraction.” International Journal of Food Properties 18, no. 8: 1625–1634. 10.1080/10942912.2014.923910.

[fsn371098-bib-0005] Greger, V. , and P. Schieberle . 2007. “Characterization of the Key Aroma Compounds in Apricots ( *Prunus armeniaca* ) by Application of the Molecular Sensory Science Concept.” Journal of Agricultural and Food Chemistry 55, no. 13: 5221–5228. 10.1021/jf0705015. 17530862

[fsn371098-bib-0006] Grosch, W. , U. C. Konopka , and H. Guth . 1992. “ACS Symposium Series.” In Characterization of Off‐Flavors by Aroma Extract Dilution Analysis, vol. 500, 266–278. American Chemical Society.

[fsn371098-bib-0007] Hou, Z. W. , Y. J. Wang , S. S. Xu , et al. 2020. “Effects of Dynamic and Static Withering Technology on Volatile and Nonvolatile Components of Keemun Black Tea Using GC‐MS and HPLC Combined With Chemometrics.” LWT ‐ Food Science and Technology 130: 109547. 10.1016/j.lwt.2020.109547.

[fsn371098-bib-0008] Huang, J. , H. T. Chen , Z. M. Zhang , Y. P. Liu , B. S. Liu , and B. G. Sun . 2021. “Investigations on the Key Odorants Contributing to the Aroma of Children Soy Sauce by Molecular Sensory Science Approaches.” Food 10, no. 7: 1492. 10.3390/foods10071492. PMC8306071 34203147

[fsn371098-bib-0009] IOFI (International Organization of the Flavor Industry) working group on methods of analysis . 2012. “IOFI Recommended Practice for the Quantitative Analysis of Volatile Flavouring Substances Using Coupled Gas Chromatography/Mass Spectrometry With Selected‐Ion Monitoring (SIM).” Flavour and Fragrance Journal 27, no. 3: 224–226.

[fsn371098-bib-0010] IOFI Working Group on Methods of Analysis . 2010. “Guidelines for Solid‐Phase Micro‐Extraction (SPME) of Volatile Flavour Compounds for Gas‐Chromatographic Analysis, From the Working Group on Methods of Analysis of the International Organization of the Flavor Industry (IOFI).” Flavour and Fragrance Journal 25, no. 6: 404–406.

[fsn371098-bib-0011] IOFI Working Group on Methods of Analysis . 2011. “Guidelines for the Quantitative Gas Chromatography of Volatile Flavouring Substances, From the Working Group on Methods of Analysis of the International Organization of the Flavor Industry (IOFI).” Flavour and Fragrance Journal 26, no. 5: 297–299.

[fsn371098-bib-0012] Ivanova‐Petropulos, V. , S. Mitrev , T. Stafilov , et al. 2015. “Characterisation of Traditional Macedonian Edible Oils by Their Fatty Acid Composition and Their Volatile Compounds.” Food Research International 77: 506–514. 10.1016/j.foodres.2015.08.014.

[fsn371098-bib-0013] Jiang, P. , Y. Liu , J. Huang , B. Fu , K. Wang , and Z. Xu . 2024. “Analysis of Volatile Flavor Compounds in Antarctic Krill Paste With Different Processing Methods Based on GC‐IMS.” Food Science & Nutrition 12: 8353–8363. 10.1002/fsn3.4425. 39479678 PMC11521673

[fsn371098-bib-0014] Li, J. Y. , Y. Dadmohammadi , and A. Abbaspourrad . 2022. “Flavor Components, Precursors, Formation Mechanisms, Production and Characterization Methods: Garlic, Onion, and Chili Pepper Flavors.” Critical Reviews in Food Science and Nutrition 62, no. 30: 8265–8287. 10.1080/10408398.2021.1926906. 34028311

[fsn371098-bib-0015] Liu, H. , Z. Y. Wang , D. Q. Zhang , et al. 2019. “Characterization of Key Aroma Compounds in Beijing Roasted Duck by Gas Chromatography‐Olfactometry‐Mass Spectrometry, Odor‐Activity Values, and Aroma‐Recombination Experiments.” Journal of Agricultural and Food Chemistry 67, no. 20: 5847–5856. 10.1021/acs.jafc.9b01564. 31042865

[fsn371098-bib-0016] Lu, Y. H. , Y. L. Chi , Y. P. Lv , G. H. Yang , and Q. He . 2019. “Evolution of the Volatile Flavor Compounds of Chinese Horse Bean‐Chili‐Paste.” LWT ‐ Food Science and Technology 102: 131–135. 10.1016/j.lwt.2018.12.035.

[fsn371098-bib-0017] Matheis, K. , and M. Granvogl . 2016. “Characterization of Key Odorants Causing a Fusty/Musty Off‐Flavor in Native Cold‐Pressed Rapeseed Oil by Means of the Sensomics Approach.” Journal of Agricultural and Food Chemistry 64, no. 43: 8168–8178. 10.1021/acs.jafc.6b03527. 27712066

[fsn371098-bib-0018] Musakhanian, J. , J. D. Rodier , and M. Dave . 2022. “Oxidative Stability in Lipid Formulations: A Review of the Mechanisms, Drivers, and Inhibitors of Oxidation.” American Association of Pharmaceutical Scientists 23, no. 5: 151. 10.1208/s12249-022-02282-0 35596043

[fsn371098-bib-0019] Ozkara, K. T. , A. Amanpour , G. Guclu , H. Kelebek , and S. Selli . 2019. “GC‐MS‐Olfactometric Differentiation of Aroma‐Active Compounds in Turkish Heat‐Treated Sausages by Application of Aroma Extract Dilution Analysis.” Food Analytical Methods 12: 729–741. 10.1007/s12161-018-1403-y.

[fsn371098-bib-0020] Rotsatchakul, P. , S. Chaiseri , and K. R. Cadwallader . 2008. “Identification of Characteristic Aroma Components of Thai Fried Chili Paste.” Journal of Agricultural and Food Chemistry 56, no. 2: 528–536. 10.1021/jf072499n. 18163558

[fsn371098-bib-0021] Smith, S. H. 2015. “In the Shadow of a Pepper‐Centric Historiography: Understanding the Global Diffusion of Capsicums in the Sixteenth and Seventeenth Centuries.” Journal of Ethnopharmacology 167: 64–77. 10.1016/j.jep.2014.10.048. 25446579

[fsn371098-bib-0022] Song, H. L. , and J. B. Liu . 2018. “GC‐O‐MS Technique and Its Applications in Food Flavor Analysis.” Food Research International 114: 187–198. 10.1016/j.foodres.2018.07.037. 30361015

[fsn371098-bib-0023] Sun, J. , M. J. Ma , B. G. Sun , et al. 2021. “Identification of Characteristic Aroma Components of Butter From Chinese Butter Hotpot Seasoning.” Food Chemistry 338: 127838 Wiley. 32822905 10.1016/j.foodchem.2020.127838

[fsn371098-bib-0024] Sun, J. , B. G. Sun , F. Z. Ren , H. T. Chen , N. Zhang , and Y. Y. Zhang . 2020. “Characterization of Key Odorants in Hanyuan and Hancheng Fried Pepper, (*Zanthoxylum bungeanum*) Oil.” Journal of Agricultural and Food Chemistry 68, no. 23: 6403–6411. 10.1021/acs.jafc.0c02026. 32423215

[fsn371098-bib-0025] Thurer, A. , and M. Granvogl . 2016. “Generation of Desired Aroma‐Active as Well as Undesired Toxicologically Relevant Compounds During Deep‐Frying of Potatoes With Different Edible Vegetable Fats and Oils.” Journal of Agricultural and Food Chemistry 64, no. 47: 9107–9115. 10.1021/acs.jafc.6b04749. 27806575

[fsn371098-bib-0026] Vidal, A. M. , S. Alcala , A. De Torres , M. Moya , J. M. Espinola , and F. Espinola . 2019. “Fresh and Aromatic Virgin Olive Oil Obtained From Arbequina, Koroneiki, and Arbosana Cultivars.” Molecules 24, no. 19: 3587. 10.3390/molecules24193587. 31590381 PMC6804064

[fsn371098-bib-0027] Wang, B. , W. Wu , J. Liu , et al. 2023. “Flavor Mystery of Spicy Hot Pot Base: Chemical Understanding of Pungent, Numbing, Umami and Fragrant Characteristics.” Trends in Food Science & Technology 139: 104137.

[fsn371098-bib-0028] Xiang, Z. M. , X. T. Chen , Z. J. Zhao , et al. 2018. “Analysis of Volatile Components in *Dalbergia cochinchinensis* Pierre by a Comprehensive Two‐Dimensional Gas Chromatography With Mass Spectrometry Method Using a Solid‐State Modulator.” Journal of Separation Science 41, no. 23: 4315–4322. 10.1002/jssc.201800636. 30299576

[fsn371098-bib-0029] Xu, L. R. , X. Mei , G. C. Wu , E. Karrar , Q. Z. Jin , and X. G. Wang . 2022. “Inhibitory Effect of Antioxidants on Key Off‐Odors in French Fries and Oils and Prolong the Optimum Frying Stage.” LWT ‐ Food Science and Technology 162: 113417.

[fsn371098-bib-0030] Yang, P. , H. L. Song , L. J. Wang , and H. Jing . 2019. “Characterization of Key Aroma‐Active Compounds in Black Garlic by Sensory‐Directed Flavor Analysis.” Journal of Agricultural and Food Chemistry 67, no. 28: 7926–7934. 10.1021/acs.jafc.9b03269. 31250635

[fsn371098-bib-0031] Yang, P. , Y. Y. Zheng , M. C. You , H. L. Song , and T. T. Zou . 2019. “Characterization of Key Aroma‐Active Compounds in Four Commercial Egg Flavor Sachimas With Differing Egg Content.” Journal of Food Biochemistry 43, no. 12: 13040. 10.1111/jfbc.13040. 31502280

[fsn371098-bib-0032] Ye, Z. , Z. X. Shang , M. Q. Li , et al. 2022. “Effect of Ripening and Variety on the Physiochemical Quality and Flavor of Fermented Chinese Chili Pepper (Paojiao).” Food Chemistry 368: 130797. 34399178 10.1016/j.foodchem.2021.130797

[fsn371098-bib-0033] Yu, J. , Y. R. Zhang , Q. J. Wang , et al. 2023. “Capsaicinoids and Volatile Flavor Compounds Profile of Sichuan Hotpot as Affected by Cultivar of Chili Peppers During Processing.” Food Research International 165: 112476. 10.1016/j.foodres.2023.112476. 36869489

[fsn371098-bib-0035] Yu, M. G. , T. Li , S. Y. Wan , et al. 2022. “Study of Aroma Generation Pattern During Boiling of Hot Pot Seasoning.” Journal of Food Composition and Analysis 114: 104844. 10.1016/j.jfca.2022.104844.

[fsn371098-bib-0034] Yu, M. G. , T. Li , S. Y. Wan , et al. 2023. “Sensory‐Directed Establishment of Sensory Wheel and Characterization of Key Aroma‐Active Compounds for Spicy Tallow Hot Pot Seasoning.” Food Chemistry 405: 134904. 10.1016/j.foodchem.2022.134904.

[fsn371098-bib-0036] Zhang, G. , Z. Ma , Y. Piao , et al. 2024. “Revealing the Potential of Star Anise Essential Oil: Comparative Analysis and Optimization of Innovative Extraction Methods for Enhanced Yield, Aroma Characteristics, Chemical Composition, and Biological Activities.” Food Science & Nutrition 12: 9540–9554. 10.1002/fsn3.4508. 39619989 PMC11606829

[fsn371098-bib-0037] Zhang, Q. , C. Wan , C. Z. Wang , et al. 2018. “Evaluation of the Non‐Aldehyde Volatile Compounds Formed During Deep‐Fat Frying Process.” Food Chemistry 243: 151–161. 10.1016/j.foodchem.2017.09.121. 29146322

[fsn371098-bib-0038] Zhang, Y. , H. L. Song , P. Li , J. Yao , and J. Xiong . 2017. “Determination of Potential Off‐Flavour in Yeast Extract.” LWT ‐ Food Science and Technology 82: 184–191. 10.1016/j.lwt.2017.04.030.

[fsn371098-bib-0039] Zhang, Y. F. , C. Zhen , B. X. Zhao , et al. 2023. “Comparative Characterization of Key Odorants and Aroma Profiles of Fragrant Rapeseed Oil Under Different Roasting Conditions.” Food Research International 163: 112195. 10.1016/j.foodres.2022.112195. 36596134

